# Editorial: Cell-based Therapies for Stroke: Promising Solution or Dead End?

**DOI:** 10.3389/fneur.2020.00171

**Published:** 2020-04-03

**Authors:** Johannes Boltze, Koji Abe, Andrew N. Clarkson, Oliver Detante, Pedro M. Pimentel-Coelho, Paulo H. Rosado-de-Castro, Miroslaw Janowski

**Affiliations:** ^1^School of Life Sciences, University of Warwick, Coventry, United Kingdom; ^2^Department of Neurology, Graduate School of Medicine, Dentistry and Pharmaceutical Sciences, Okayama University, Okayama, Japan; ^3^Department of Anatomy, Brain Health Research Centre and Brain Research New Zealand, University of Otago, Dunedin, New Zealand; ^4^Stroke Unit, Neurology Department, Grenoble Hospital, Grenoble, France; ^5^Grenoble Institute of Neurosciences, Inserm U1216, Université Grenoble Alpes, Grenoble, France; ^6^Instituto de Biofísica Carlos Chagas Filho, Federal University of Rio de Janeiro, Rio de Janeiro, Brazil; ^7^Instituto Nacional de Ciência e Tecnologia em Medicina Regenerativa, Rio de Janeiro, Brazil; ^8^Institute of Biomedical Sciences, Federal University of Rio de Janeiro, Rio de Janeiro, Brazil; ^9^Department of Radiology, Federal University of Rio de Janeiro, Rio de Janeiro, Brazil; ^10^D'Or Institute for Research and Education, Rio de Janeiro, Brazil; ^11^Department of Diagnostic Radiology and Nuclear Medicine, University of Maryland School of Medicine, Baltimore, MD, United States; ^12^NeuroRepair Department, Mossakowski Medical Research Centre, Polish Academy of Sciences, Warsaw, Poland

**Keywords:** stem cells, stroke, mesenchymal stem cells (MSC), brain, treatment, translational medicine, ischemia, clinical trials

The introduction of recanalization procedures has revolutionized acute stroke management, although factors such as the narrow time window, strict eligibility criteria, and logistical limitations still exclude the majority of patients from treatment. In addition, residual deficits are present in many patients who undergo therapy, preventing their return to premorbid status. Hence, there is a strong need for novel, and ideally complementary, approaches to stroke management.

In preclinical experiments, cell-based treatments have demonstrated beneficial effects in the subacute and chronic stages following stroke (1–3) and therefore are considered a promising option to supplement current clinical practice. At the same time, great progress has been made in developing clinically feasible delivery and monitoring protocols (4). However, efficacy results initially reported in clinical studies fell short of expectations (5) raising concerns that cell treatment might eventually share the “dead end fate” of many previous experimental stroke therapies. This Research Topic reviews some of the latest and most innovative studies to summarize the state of the art in translational cell treatments for stroke.

## New Mechanistic Insights from Preclinical Experiments

Umbilical cord blood (UCB)-derived cells are a widely available and rich source of relatively young cells. However, it is unclear which fraction of this heterogeneous population is responsible for the therapeutic effects reported after stroke. Gornicka-Pawlak et al. investigated CD34^−^ mononuclear cells (MNCs) either freshly prepared or cultured for 3 days vs. a UCB derived neural stem cell line. The study particularly focused on restoring cognitive functions after stroke what is a novel endpoint for the UCB derived neural stem cell line. Freshly prepared cells were found most effective, which is in line with what has been reported for motor and sensory functions using UCB-MNCs after stroke (6). An enriched environment was provided to the animals, further fostering cognitive recuperation in a clinically meaningful setup. Mu et al. revealed that a combination of adipose stem cells and rehabilitation after experimental stroke is beneficial. This approach follows the newest STem Cells as an Emerging Paradigm in Stroke (STEPS) recommendations and is expected to provide more translationally relevant data (7). Hwang et al. proved that a combination of UCB-MNC and erythropoietin is also beneficial. Green et al. stereotaxically applied neural stem cells in the subacute stage after large cortico-striatal and smaller striatal strokes. Cell graft vitality was better preserved in smaller, striatal lesions, which are associated with a stabilization of functional neuronal networks. However, this effect was only transient, indirectly pointing to other long-term degenerative mechanisms and processes that thus far have not been identified. Encouraging results were reported regarding the efficacy of bone marrow-derived mesenchymal stem cells (MSCs) which have been applied in numerous preclinical trials for almost two decades. Satani et al. performed a systematic review and meta-analysis on 141 preclinical studies, confirming robust efficacy in acute and subacute time windows. It is noteworthy that comparable effects were seen in multiple labs around the world. Based on these robust data, the authors suggest that this approach should advance to carefully planned and implemented clinical trials.

## Translational and Clinical Considerations

Defining the best-suited cell source is crucial to taking the translational process from the preclinical to the clinical stage. Ideally, the respective cells should be applicable for autologous and allogeneic use, and should exert beneficial effects via indirect (“bystander”) effects while also exhibiting the potential for replacement of brain cells including astrocytes, oligodendrocytes and, most challenging, neurons thus covering all potential aspects of brain tissue regeneration (8). Recent research by Gancheva et al. revealed that dental pulp stem cells may perfectly fill this role. Another relevant aspect to translation is the safety of cell applications. Potential adverse events, such as secondary microinfarction, were reported when intraarterially administering large diameter cell populations such as MSCs. However, this phenomenon seems to depend on infusion speed and, in particular, cell dose, since lower doses can be safely delivered to the brain (9, 10). Cell engineering is another approach used to mitigate these potential adverse effects, for instance by increasing cell egress from cerebral capillaries (11). Moreover, no strong evidence of such complications has been observed after MSC delivery in clinics (12). The use of MSC-derived extracellular vesicles in place of MSCs also may help circumvent this problem. Bang and Kim, both working at the forefront of clinical translation, summarize the state of the art in this field, focusing on emerging clinical applications and remaining challenges.

Results from clinical cell therapy studies in stroke have been reported for intravenous injections (13, 14) and intracerebral grafts (15). Although overall safety has been confirmed, analysis of efficacy endpoints suggests that magnitude of effect may be smaller in human than animal studies, and a number of logistical challenges also have been identified. Krause et al. reviewed such problems, providing an unbiased overview of bottlenecks in the translational process, and discussing relevant aspects such as cost-to-benefit ratios and the role of industry-driven clinical research. Despite the moderate collective tepid enthusiasm regarding cell-based approaches, encouraging clinical data is available. Haque et al. report metabolic changes observed by magnetic resonance spectroscopy in the brains of patients being treated with autologous bone marrow-derived MNCs. These changes correlated with NIHSS scores and might not only indicate efficacy, but could also be used as surrogate markers for treatment efficacy in future clinical trials.

## Summary and Outlook

Although clinical translation of cell-based therapies is clearly gaining momentum, a number of open questions remain. One is the role of co-morbidities, which are abundantly present in human patients but are rarely modeled preclinically. Laso-Garcia et al. have analyzed this discrepancy and provide a comprehensive summary on effects of the most relevant comorbidities including hypertension, diabetes, and obesity both from clinical and preclinical perspectives. Aspects such as potential cell-drug interactions also await clarification (16). Finally, remarkable developments toward precision stem cell medicine have been achieved, which may facilitate stem cell-based therapies. Stem cell labeling and real-time imaging are capable of improving precision of transplantations (17). Progress in biomarker research (18) and artificial intelligence (19) may soon revolutionize research on outcome assessment, which will be pivotal to the future success of stem cell therapies. In summary, the road on which we travel with cell therapies for stroke is probably not a dead end but the journey remaining is challenging and long. Nevertheless, the overall research progress may finally shed light on the path, with this Research Topic identifying some of the most important past and future milestones along the way.

## Author Contributions

All authors listed have made a substantial, direct and intellectual contribution to the work, and approved it for publication.

### Conflict of Interest

The authors declare that the research was conducted in the absence of any commercial or financial relationships that could be construed as a potential conflict of interest.
